# Comprehensive comparison of Apple Watch and Fitbit monitors in a free-living setting

**DOI:** 10.1371/journal.pone.0251975

**Published:** 2021-05-26

**Authors:** Yang Bai, Connie Tompkins, Nancy Gell, Dakota Dione, Tao Zhang, Wonwoo Byun

**Affiliations:** 1 Department of Health, Kinesiology, and Recreation, University of Utah, Salt Lake City, UT, United States of America; 2 Department of Rehabilitation and Movement Science, University of Vermont, Burlington, VT, United States of America; 3 Department of Physical Therapy, Arcadia University, Glenside, PA, United States of America; 4 Department of Kinesiology, Health Promotion, and Recreation, University of North Texas, Denton, Texas, United States of America; Linneaus University, SWEDEN

## Abstract

**Objectives:**

The aim of this study was to evaluate the accuracy of three consumer-based activity monitors, Fitbit Charge 2, Fitbit Alta, and the Apple Watch 2, all worn on the wrist, in estimating step counts, moderate-to-vigorous minutes (MVPA), and heart rate in a free-living setting.

**Methods:**

Forty-eight participants (31 females, 17 males; ages 18–59) were asked to wear the three consumer-based monitors mentioned above on the wrist, concurrently with a Yamax pedometer as the criterion for step count, an ActiGraph GT3X+ (ActiGraph) for MVPA, and a Polar H7 chest strap for heart rate. Participants wore the monitors for a 24-hour free-living condition without changing their usual active routine. MVPA was calculated in bouts of ≥10 minutes. Pearson correlation, mean absolute percent error (MAPE), and equivalence testing were used to evaluate the measurement agreement.

**Results:**

The average step counts recorded for each device were as follows: 11,734 (Charge2), 11,922 (Alta), 11,550 (Apple2), and 10,906 (Yamax). The correlations in steps for the above monitors ranged from 0.84 to 0.95 and MAPE ranged from 17.1% to 35.5%. For MVPA minutes, the average were 76.3 (Charge2), 63.3 (Alta), 49.5 (Apple2), and 47.8 (ActiGraph) minutes accumulated in bouts of 10 or greater minutes. The correlation from MVPA estimation for above monitors were 0.77, 0.91, and 0.66. MAPE from MVPA estimation ranged from 44.7% to 55.4% compared to ActiGraph. For heart rate, correlation for Charge2 and Apple2 was higher for sedentary behavior and lower for MVPA. The MAPE ranged from 4% to 16%.

**Conclusion:**

All three consumer monitors estimated step counts fairly accurately, and both the Charge2 and Apple2 reported reasonable heart rate estimation. However, all monitors substantially underestimated MVPA in free-living settings.

## 1. Introduction

Historically, activity monitors have been used in research settings to provide objective measurement of physical activity (PA) [1]. Consumer PA monitors, such as Apple Watch and Fibit, gained popularity among consumers. The consumer PA monitors collect a variety of PA metrics, including distance traveled, step count, sedentary breaks, intensity of activity, heart rate, and sleep tracking [2–4]. As a result of their ubiquity, consumer monitors are also now often utilized by researchers in PA behavior change interventions [5,6]. Consumer PA monitors and their associated apps and/or websites incorporate some aspects of behavior change theories and techniques as well as design features including goal-setting, self-monitoring, feedback, social support, shaping knowledge, repetition and substitution and rewarding. In a critical analysis, the average number of behavior change techniques adopted was 16.3 across seven consumer monitors reviewed [7].

Furthermore, these consumer monitors can be used as a tool for promoting PA with a tailored approach as feedback is personalized to address individual, behavioral and physiological characteristics with respect to PA [8]. Fitbit products and Apple Watch wearables are the most popular brands and have the biggest shares in the wearable market. Numerous intervention studies have also utilized consumer monitors to assess and promote PA [6,9–11]. According to ClinicalTrials.Gov, a search for ‘Fitbit/Apple watch,’ with ‘interventional’ as study type, indicated more than 400 clinical trials used- or are currently using Fitbit products and 26 trials used Apple watches. In those intervention studies, Fitbit has been used along with text messages [12], goal-setting [12], a web-based lifestyle program [13], lifestyle behavior change education sessions [14], a structured exercise intervention [15], counseling [12], a monetary incentive [16] and a support group [17,18]. It has also been widely used in clinical populations such as adolescents with attention deficit hyperactivity disorder [17], adolescent and young adult cancer survivors [18], adults with serious mental illness [7], type 2 diabetes [14], older adults with high risk of coronary heart disease [15], and people with Down Syndrome [19].

Extensive validation studies have been conducted in controlled settings with Fitbit products and literature to date has shown relatively high accuracy in estimating steps and heart rate [19]. However, there is little to support the most popular consumer monitors (i.e., newer Fitbit products and Apple Watch) in estimating PA metrics under free-living settings. Therefore, validation of consumer monitors under free-living settings is needed to provide empirical evidence in utilizing activity monitors as tools in monitoring and facilitating exercise behavior changes. Responding to this research gap in the literature, the aim of this study was to examine the validity of steps, moderate-to-vigorous physical activity (MVPA) minutes, and heart rate of three popular consumer-grade activity monitors in a 24-hour free-living setting. Our hypothesis was that the steps and heart rate measured by consumer monitors are equivalent to the criterion measures but not for MVPA estimation.

## 2. Methods

### Sample and procedures

A total of 52 participants were enrolled into the study. Four participants did not complete data collection and were therefore excluded from the paper. The inclusion criterion were 1) healthy adults; 2) aged 18–59 years; 3) willingness to wear multiple monitors simultaneously for 24 hours. A phone screening was completed for each participant and included a standard clinical exercise screening tool, the PA Readiness Questionnaire with additional questions to exclude participants 1) requiring mobility assistance; 2) at risk for adverse events with PA including heart condition, chest pain without PA dizziness, bone or joint problem; 3) taking medication for hypertension/heart condition; 4) with metal allergy and tattoos on either wrist [20]. The participants were instructed on wearing six different PA monitors for a full day (24 hours) except while sleeping or bathing, and returned the monitors the next day. A recording sheet was also provided for them to write down the time they placed the monitors on and off and record the number of steps on the pedometer display. Participants were asked to adhere to normal daily activities. When participants returned to the lab at a convenient, pre-scheduled time the following day, trained research assistants synced the three consumer monitors with their respective mobile applications to retrieve the previous day steps and active minutes. The study protocol was approved by the university’s Institutional Review Board. Informed consent forms were obtained prior to data collection.

### Measures

#### Anthropometric, demographic and clinical measures

Anthropometrics including stature, body mass, and percent body fat were obtained in a private room and measured by the InBody 570 (InBody, Cerritos, CA, USA). Demographics including gender and age data were collected and entered into the associated smart phone/tablet application to initialize the monitors. The smart phone and tablet were owned by the researchers. Thus, the ownership of smart phone/tablet was not part of eligibility criterion. Blood pressure and resting heart rate were measured with the Omron 10 Series, a blood pressure cuff (Omron Healthcare, Inc, Hoffman Estates, IL, USA) after participants had been seated and rested for at least 10 minutes.

#### Criterion measures

Three criterion monitors were used to validate steps, MVPA, and heart rate separately. ActiGraph GT3X+ (ActiGraph, Pensacola, FL) (ActiGraph) was worn on the right hip anterior to the iliac crest as a criterion to measure MVPA. The participant was asked to fasten accelerometer tightly to the belt. ActiGraph is one of the most frequently used criterion measurement to validate other monitors in research setting and has been widely used in clinical trials [21]. The participant was asked to fasten accelerometer tightly to the belt. A Yamax Digi-Walker SW-200 (Yamax), calibrated prior to data collection, was worn on the waistband anterior to the left iliac crest as criterion measure of steps. No differences between Yamax compared with actual steps taken were observed at walking speeds of 2.5–4 miles per hour in prior validation study [22–24]. A Polar H7 HR sensor chest strap was worn on the chest as the criterion measure of heart rate. Polar H7 had almost perfect correlations with ECG (R = 1.00 [0.99; 1.00]) in a recent validation study [25]. The ActiGraph data were downloaded with ActiLife software and processed using the Sojourn method to estimate the daily MVPA as criterion for active minutes. The Sojourn method uses a machine-learning approach and it has improved the accuracy and precision in estimating free-living MET-hours compared to other traditional accelerometer data process [21]. It was unclear as to whether the bout criterion was used in the Fitbit or Apple Watch products; therefore, we used two criterion outcomes from ActiGraph, one with a 10-minute bout filter and the other one without a bout filter [26,27]. The criterion of daily steps from Yamax was obtained from the data recording sheet that participants completed at the end of their monitor wear. For heart rate, the compatibility between ActiGraph and the criterion data from the Polar H7 allowed for Bluetooth download of minute-by-minute data through ActiLife software.

#### Comparison measures

Participants concurrently wore an Apple Watch series 2 (Apple Inc., Cupertino, CA) on the left wrist and Fitbit Alta and Fitbit Charge 2 (Fitbit Inc., San Francisco, CA) on their right wrist. The placement of the monitors were consist across participants. The estimates of daily steps and active minutes from the consumer monitors were obtained directly from the smart phone/applications. The Fitbit Charge 2 heart rate data was downloaded through a third-party platform, Fitabase (Small Steps Labs LLC., San Diego, CA). The Apple Watch 2 heart rate data was accessed through Apple Health Kit. Because the heart rate data from Apple Watch 2 is not captured at a fixed interval, the heart rate data from Polar H7 HR sensor was merged separately with the Apple Watch 2 and Fitbit Charge 2 at the minute level using timestamps. The number of minutes available for heart rate data were different from Fitbit Charge 2 and Apple Watch 2. Heart rate data was examined at three intensity levels of PA: sedentary behavior (SB), light PA (LPA), and MVPA. The intensity level was determined by ActiGraph data estimation and metabolic equivalents (METs) that SB is METs < 1.5, 1.5 –<3 METs for LPA, and MVPA of ≥3 METs [28].

### Statistical analyses

Descriptive statistics were used to summarize the participants’ characteristics including age, stature, body mass, body mass index (BMI), percent of body fat, systolic and diastolic blood pressure, and resting heart rate. To qualify the measurement errors, the following indicators were calculated between the monitors’ estimation (Fitbit Alta, Fitbit Charge 2, and Apple Watch 2) and criterion measures (Yamax, ActiGraph, and Polar H7) in steps, MVPA, and heart rate: group-level agreement indicators of mean bias and mean percentage error (MPE), individual-level agreement indicators of mean absolute percentage error (MAPE), and root mean square error (RMSE). Mean bias was calculated by averaging the difference between the criterion and the estimate (i.e., Steps_criterion_- Steps_monitors_). MPE computed the error as a percentage deviation from the criterion to standardize the error (i.e., average steps difference from two measures divided by Steps_criterion_). MAPE expressed the absolute value of the EE difference before dividing by the Steps_criterion._ RMSE was the quadratic mean of the difference between the criterion and the estimate. Bland-Altman plot, Pearson product-moment correlation, and equivalence testing were also used to evaluate the overall agreement of the consumer monitors with the criterion measurements [29]. Equivalence testing reversed the traditional null and alternative hypotheses along with the conclusions are inverted. We tested the null hypothesis: there is a difference between the criterion and the consumer monitor. Therefore, rejection of the null hypothesis indicates the two methods are equivalent with each other, statistically. The next step was to set up the equivalence zone, although no guidelines exist to define the best equivalence zone. Thus, it is usually based on prior evidence or on the practical or clinical meaning of the value. We used ±10% of the mean of criterion measures as the equivalence zone based on a series of previous studies [3,4]. We tested whether the 90% confidence interval from the consumer monitor measurement would fall within the equivalence zone with 95% precision (α was set up at 0.05). The detailed process with applied examples were described in a methodology paper [30]. Multiple statistical indicators were calculated in order to provide a comprehensive overview of the measurement error. The measurement agreement conclusion was made primarily based on MAPE that is less than 20% and whether the consumer monitor measurement would fall within the equivalence zone.

## 3. Results

**Fig 1:**
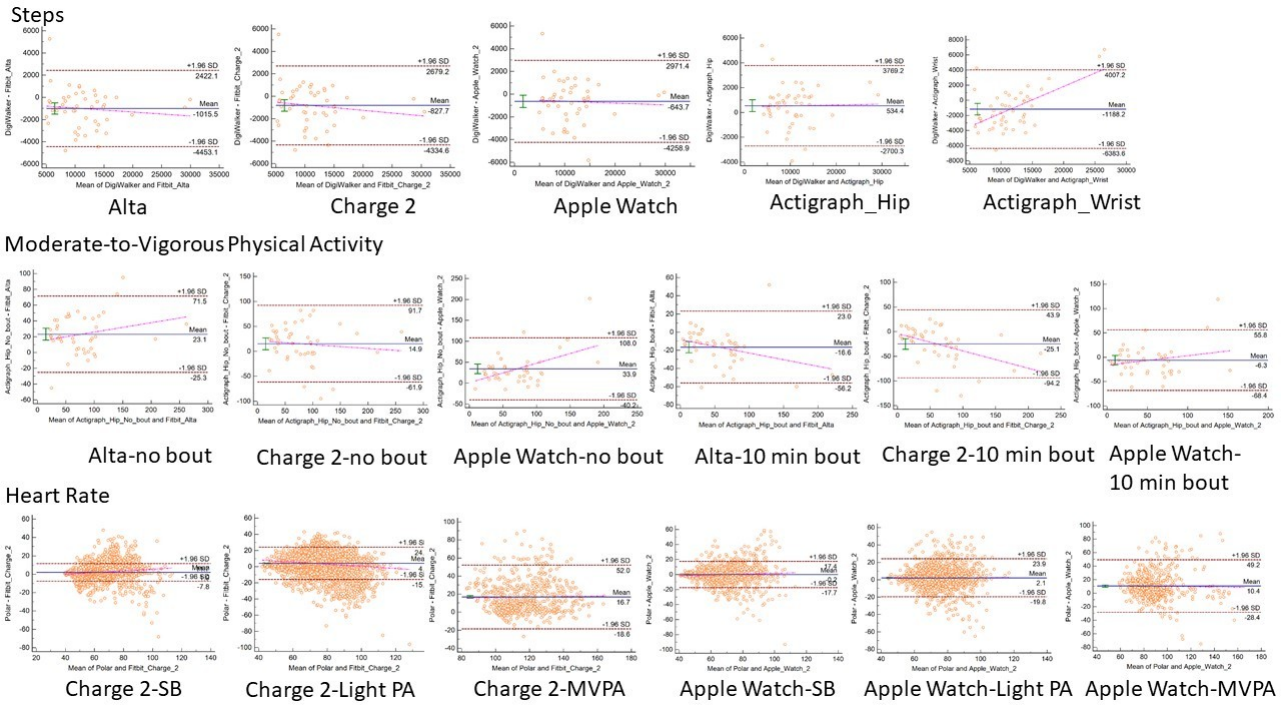
Agreement between criterion and comparison monitors on 95% equivalence testing.

**Fig 2:**
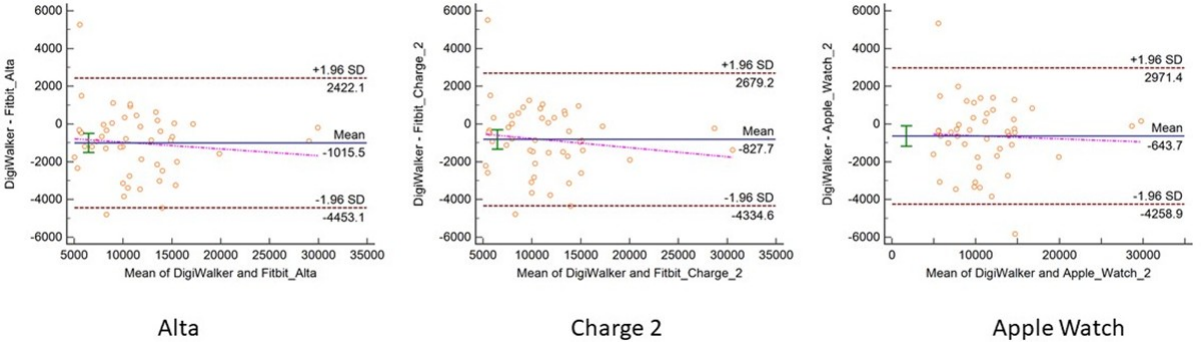
Bland-Altman plots comparing steps between Yamax and research and consumer activity monitors.

**Fig 3:**
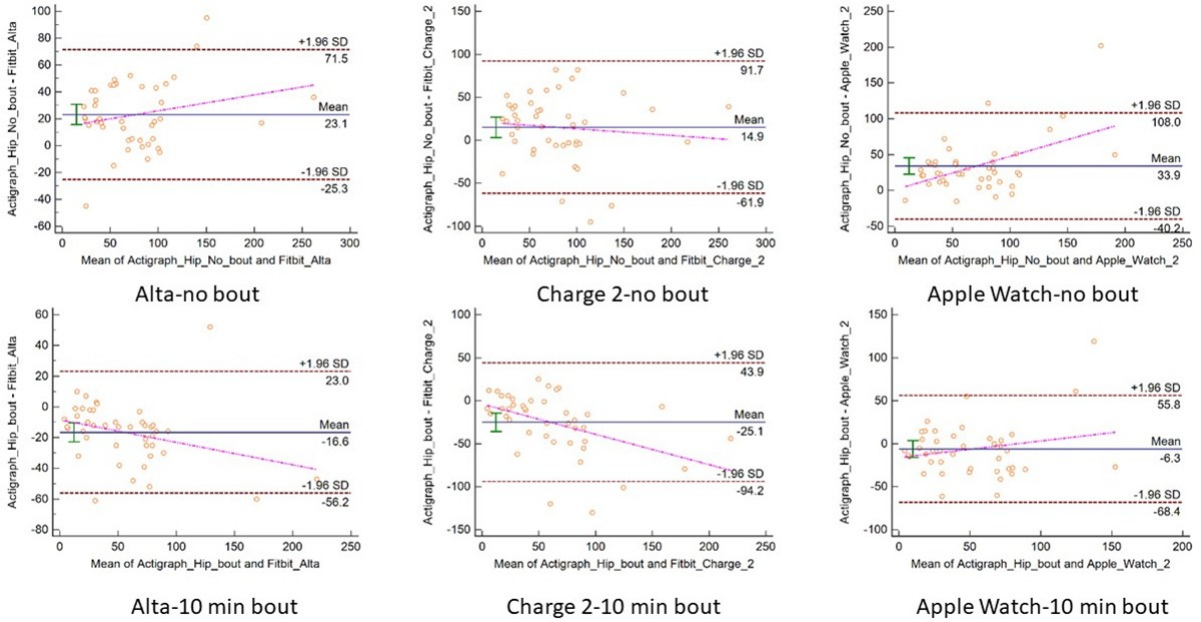
Bland-Altman plots comparing moderate-to-vigorous physical activity between ActiGraph and three consumer activity monitors with- and without bout criterion.

**Fig 4:**
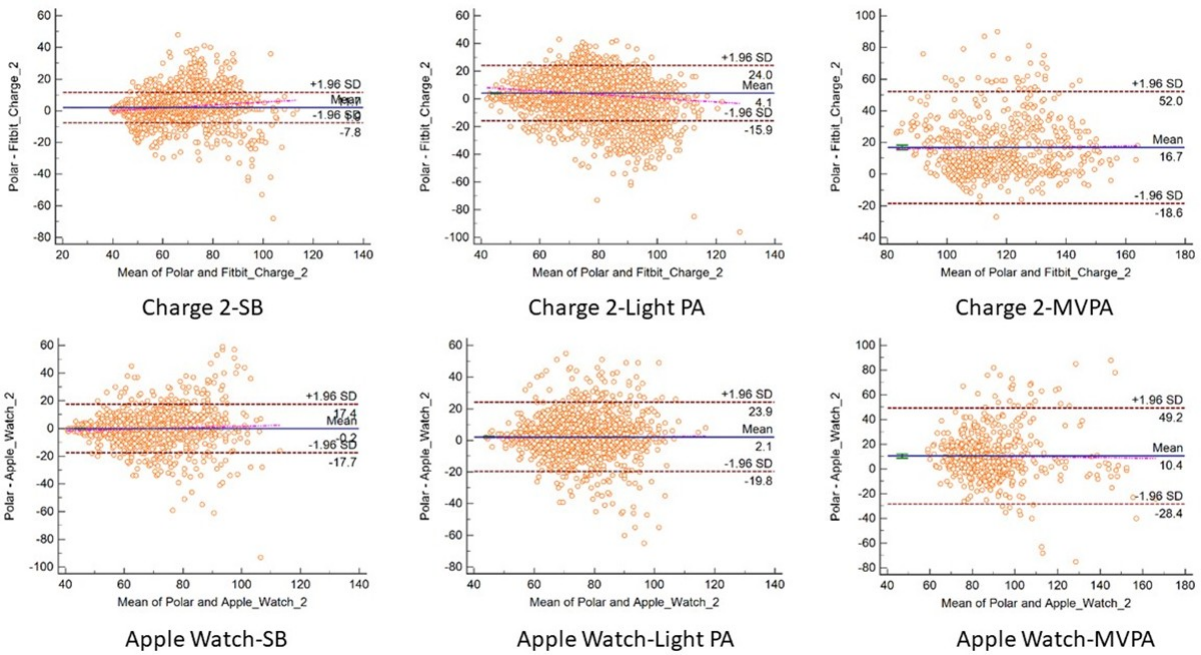
Bland-Altman plots comparing heart rate between Polar H7 and Apple Watch 2 and Fitbit Charge 2.

The participant demographic and anthropometric characteristics are reported in Table 1. The sample consisted of a group of 48 young (mean age = 26.8±3.0) and healthy participants of 31 females and 17 males. The measurement error indicators between consumer monitors and criterion measures are presented for daily steps, daily MVPA, and heart rate by intensity in Tables 2 and 3. The equivalence testing results for steps, MVPA, and heart rate are presented in Fig 1. The Bland-Altman plots examining the measurement agreement between consumer activity monitors to criterion measures about the three metrics are presented in Figs 2–4.

**Table 1:** Descriptive demographics of the participants (*N* = 48).

	Mean	Standard Deviation	Minimum	Maximum
Age (years)	26.8	11.9	18.0	59.0
Stature (cm)	168.4	10.4	147.0	190.5
Body mass (kg)	66.3	13.0	42.6	105.4
Body Mass Index (kg/m^2^)	23.2	3.0	17.9	32.9
Body Fat Percentage	20.5	8.5	7.7	36.1
Systolic Pressure (mmHg)	112.1	13.5	87.7	147.0
Diastolic Pressure (mmHg)	70.6	9.6	53.7	95.0
Heart Rate (bpm)	78.8	49.3	49.0	93.7

**Table 2:** Validity of steps and moderate-to-vigorous minutes estimation from three consumer monitors and two research monitors (*N* = 48).

	Mean (SD)	MPE (SD)	MAPE (SD)	RMSE	Correlation
Steps/day					
Yamax SW-200	10906 (5166)				
Fitbit Alta	11922 (5348)	-15.2% (35.2%)	20.7% (32.2%)	2141	0.94**
Fitbit Charge 2	11734 (5413)	-10.3% (23.4%)	17.1% (18.9%)	1989	0.94**
Apple Watch 2	11550 (5248)	-11.8% (33.2%)	20.3% (28.7%)	2055	0.94*
MVPA without 10 minutes bout in minutes/day					
ActiGraph	87.1 (52.2)				
Fitbit Alta	64.0 (46.9)	-26.0% (366.7%)	92.3% (355.5%)	32	0.90**
Fitbit Charge 2	72.2 (56.0)	-35.8% (319.0%)	91.0% (307.5%)	42	0.74**
Apple Watch 2	53.3 (33.5)	-21.4% (120.3%)	58.9% (106.7%)	48	0.67**
MVPA with 10 minutes bout in minutes/day					
ActiGraph	47.8 (41.1)				
Fitbit Alta	64.0 (46.9)	32.0% (50.0%)	44.7% (38.7%)	26	0.91**
Fitbit Charge 2	72.2 (56.0)	50.1% (88.9%)	67.3% (76.3%)	45	0.77**
Apple Watch 2	53.3 (33.5)	26.2% (74.6%)	55.4% (55.7%)	31	0.66**

Note: MPE = Mean Percent Error; MAPE = Mean Absolute Percent Error; RMSE = Root Mean Square Error; SD = Standard Deviation

** Correlation is significant at an alpha level of 0.01 (2-tailed)

* Correlation is significant at an alpha level of 0.05 (2-tailed).

Equivalence Zone from criterion measure was underlined.

**Table 3:** Validity of heart rate estimation from Apple Watch 2 and Fitbit Charge 2.

	No. of minutes	Polar Mean (SD)	Monitor Mean (SD)	MPE (SD)	MAPE (SD)	RMSE	Correlation
**Fitbit Charge 2**							
Sedentary	7811	61.5 (11.5)	59.6 (10.6)	2.7% (7.1%)	4% (6.1%)	8.94	0.90**
Light PA	6559	74.8 (12.2)	70.7 (13.7)	4.9% (14%)	10% (10%)	11.34	0.70**
MVPA	618	125.6 (18.0)	108.9 (17.7)	13% (13%)	14% (12%)	22.33	0.49**
**Apple Watch 2**							
Sedentary	2444	66.6 (12.3)	66.7 (11.8)	-1.1% (13%)	7% (11%)	5.33	0.73**
Light PA	2075	75.6 (12.0)	73.5 (11.8)	1.8% (15%)	10% (11%)	10.97	0.56**
MVPA	590	94.3 (19.3)	83.9 (19.7)	9.6% (19%)	16% (14%)	24.54	0.49**

Note: PA = Physical Activity; MVPA = Moderate to Vigorous Physical Activity; MPE = Mean Percent Error; MAPE = Mean Absolute Percent Error; RMSE = Root Mean Square Error; SD = Standard Deviation.

** Correlation is significant at an alpha level of 0.01 (2-tailed).

*Correlation is significant at an alpha level of 0.05 (2-tailed).

For daily steps, all other monitors overestimated the number of steps compared to Yamax. All three consumer monitors had a MAPE of ~20% and similar RMSE of about 2,000 steps per day. Although the correlations between Yamax and the consumer-grade monitors were high (r = 0.94), all of the devices were out of the equivalence testing zone (Fig 1). Steps measured from the Apple Watch 2 was the most aligned with the equivalence testing zone established by Yamax (see Table 2). None of the monitors showed evident patterns of proportional systematic bias against Yamax. The Bland-Altman plots revealed the narrowest 95% limits of agreement for Fitbit Alta (see Fig 2).

For MVPA, without the bout filtering, all three consumer monitors underestimated the MVPA minutes with a MPE of 20% to 35% and MAPE over 50% to 90% when compared to ActiGraph. The RMSE was also high and ranged from 32 to 48 minutes a day. Although the correlation was statistically significant, the strength varied from 0.67 with Apple Watch 2 to 0.90 with Fitbit Alta. Fig 1 indicated that none of the monitors fell in the equivalence testing zone. The Bland-Altman plots from all three monitors showed evident patterns of proportional systematic bias. Both of the Fitbit products tended to overestimate more when PA duration increased. On the contrary, Apple Watch 2 tended to overestimate MVPA when PA duration was low and underestimate MVPA when PA duration became high (see Fig 3). The measurement error was smaller with the bout filter added during processing the ActiGraph data with Apple Watch 2 having the closest average MVPA estimation. The MPE and MAPE remained high for Fitbit Charge 2 with up to 50% MPE and 67% MAPE whereas the other monitors were lower with the Apple Watch 2 with 26.2% MPE and Fitbit Alta with 44.7% MAPE. The Apple Watch 2 fell close to the equivalence testing zone while the estimation from the other two devices were much further from the equivalence testing zone (see Table 2). The Bland-Altman plots showed that the Fitbit Alta and Apple Watch 2 underestimated more when overall activity amount increased. The Fitbit Alta had the narrowest 95% limits of agreement regardless of bout filter (see Fig 3).

Both Fitbit Charge 2 and Apple Watch 2 estimated heart rate most accurately during sedentary periods with MPE <3% and MAPE <7%, followed by light PA with <5% MPA and 10% of MAPE. For MVPA, both Apple Watch 2 and Fitbit Charge 2 had similar measurement errors of 16% and 14% of MAPE in estimating heart rate. Heart rate estimation from Fitbit Charge 2 and Apple Watch 2 fell in the equivalence testing zone during SB and light PA but not MVPA. The correlation between monitors and criterions was highest among sedentary periods, followed by light PA, and then MVPA period for both Apple Watch 2 and Fitbit Charge 2 (see Table 3). The distribution of error in Bland-Altman plots indicated no proportional systematic bias in either Fitbit Charge 2 or Apple Watch 2 heart rate estimation across three intensities. Fitbit Charge 2 showed slightly narrower 95% limits of agreement than Apple Watch 2 in three intensities.

## 4. Discussion

The results of this study revealed low to acceptable validity from three popular consumer monitors, Apple Watch 2, Fitbit Charge 2 and Fitbit Alta, in free-living settings in estimating steps, MVPA, and heart rate. The overall error in steps was ~ 20% while error in MVPA ranged from 45% up to 90%. The monitors were most accurate in estimating heart rate with a measurement error of 4% to 16%. The current study adds informative evidence on the accuracy of consumer monitors under free-living settings.

In recent studies, steps have been the most widely evaluated metric in free-living settings. The current study found a MAPE up to 20% in the three consumer monitors over a 24-hour free-living evaluation and all overestimated the steps. Results from the current study align well with several other studies that assessed Fitbit products and also found an overestimation of steps [31–34]. Tedesco et al. reported a MAPE of 17.1% with the Fitbit Charge 2 which is incidentally the exact same level MAPE as this study found [35]. Collins et al. also found the Fitbit Charge 2 overestimated steps; although they reported higher measurement error with an overestimation of 39% [36].

Energy expenditure (EE), METs and MVPA appear to be the most challenging metrics to estimate [29]. Although both Fitbit Charge 2 and Apple Watch 2 showed acceptable accuracy in estimating heart rate, there was no apparent evidence to support that the combination of accelerometer and heart rate technology (or the consumer monitors manufactures actually used both accelerometer and heart rate data to estimate MVPA) could increase the accuracy of estimating MVPA [27]. Collins and colleagues reported 75% MAPE, which falls within the range of the findings in the current study, with- and without a bout requirement, 67.3% and 91.0%, respectively [36]. Moreover, Tedesco et al. found the Fitbit Charge 2 overestimated MVPA 12.6 minutes per day in older adults [35]. Findings from review studies that examined other versions of Fitbit products were mixed. For instance, Driscoll reviewed 60 studies validating EE from both consumer and research monitors and concluded that EE estimates vary in accuracy depending on activity type. Among all the monitors reviewed, no significant differences were found between Apple Watch, Fitbit Charge HR, and Fitbit Flex from criterion measure [37]. Another systematic review, that included eight studies evaluating Fitbit devices in free-living settings, reported that the Fitbit devices were likely to overestimate time spent in higher-intensity activity and unlikely to provide accurate measures for EE in any testing condition. However, the criterion for error they set up for free-living setting was 10% [19]. No validation studies that assessed the Apple Watch 2 in free-living settings were identified. It should be noted that that there was variability in the choice of MVPA cut-offs and algorithms for processing the ActiGraph data, which could contribute to the mixed findings.

The findings of this study revealed that both Apple Watch 2 and Fitbit Charge 2 were found to have acceptable validity of heart rate measurements under free-living settings, especially for SB and light PA. To our knowledge, this is one of the first studies that assessed heart rate validity in free-living settings for all three monitors. Gorny and colleagues evaluated data from Fitbit Charge HR consumer monitors in free-living conditions [38]. The only statistical validity indicator reported was intraclass correlation coefficients (ICCs) which included an overall ICC of 0.83, similar to this study. They also found the Fitbit Charge HR underestimated heart rate in both low and high intensity PA; however, the results of our study indicated that Fitbit Charge 2 overestimated heart rate in three PA intensities. The discrepancy may be attributable to changes in the product between the original version of Fitbit Charge and the updated Charge 2 used in our study.

It is not surprising that higher measurement errors are found in free-living conditions than controlled lab settings. Several studies have been conducted in lab settings to validate the Fitbit Charge 2 and Apple Watch 2 in estimating steps, heart rate, and EE. A variety of exercise modules and free-living activities were designed to validate the feature of estimating heart rate. Several studies validated heart rate during cycling [39–41] with findings indicating an underestimation of heart rate from Fitbit Charge 2 and Apple Watch 2 displaying the greatest validity with heart rate. As exercise intensity increased, there was greater underestimation of heart rate [39,41]. Xie et al. evaluated the validity in estimating steps during walking, running and cycling in a lab setting. They found the Apple Watch 2 had the highest MAPE of 42% among all of the monitors they examined [40]. No other studies were identified that evaluated Fitbit Charge 2 or Alta for estimating steps in the lab setting. EE estimation compared to metabolic cart readings was examined in several controlled studies and revealed varied measurement errors across different monitors and different exercise modes. Xie et al. reported MAPE of < 10% in running, close to 20% in cycling, and around 45% in walking from Apple Watch 2 estimating EE (40). Boudreaux and colleagues found Fitbit Charge 2 underestimated EE (MAPE = 75%) and Apple Watch 2 overestimated EE (MAPE = 21%) in cycling [39]. The findings of the current study showed very comparable results for steps and heart rate to monitors validated under lab settings but the EE was much larger.

This study is not without limitations. Participants included in the current study were healthy and mostly young adults. Additional research is needed to assess validity of the monitors in other special populations, particularly in those without a typical locomotive pattern. Another limitation is the difference in sample size between Apple Watch 2 and Fitbit Charge 2, in estimating heart rate due to the different epochs used in monitors to export the data. Fitbit provides minute-by-minute heart rate data while Apple Watch 2 data output was dependent on how frequently the users changed their behaviors and/or intensity. The trend of PA wearable devices to be worn on the wrist brings about the concern of potential spurious results from upper extremity movement. This study did not capture the potential of activities involving prolonged wrist movements, which may impact the accuracy of wrist-worn devices [42,43]. Although we provided a picture of the monitors’ placement to participants during the 24 hours monitoring, there is no way to guarantee that the participants put them back in the correct manner, which could potentially impact the accuracy of the monitors. Lastly, how the consumer monitor companies process the raw data and the algorithm are remained unknown. There might be discrepancies on how to define and classify the MVPA between consumer monitors and the science community.

In conclusion, the findings of this study showed acceptable validity for estimating heart rate and steps but poor validity for MVPA in three types of consumer monitors. Data from the Apple Watch 2, Fitbit Charge 2, and Fitbit Alta, should be interpreted and used with caution, especially with higher intensity of exercise. As companies releasing new consumer activity tracking devices do not generally release the method for calculations on steps, heart rate, and EE, among others, researchers will need to continue to evaluate the efficacy of current devices in their ability to provide accurate information to consumers.

## Supporting information

S1 Data (CSV)
